# Studying the association between musculoskeletal disorders, quality of life and mental health. A primary care pilot study in rural Crete, Greece

**DOI:** 10.1186/1471-2474-10-143

**Published:** 2009-11-20

**Authors:** Maria D Antonopoulou, Athanasios K Alegakis, Alexander G Hadjipavlou, Christos D Lionis

**Affiliations:** 1 Clinic of Social and Family Medicine, Department of Social Medicine, School of Medicine, University of Crete, 74100 Heraklion, Crete, Greece; 2 Spili Health Centre, Health and Welfare Region of Crete, 74053 Spili, Crete, Greece; 3 Department of Orthopaedics and Traumatology, School of Medicine, University of Crete, 74100 Heraklion, Crete, Greece

## Abstract

**Background:**

The burden of musculoskeletal disorders (MSD) on the general health and well-being of the population has been documented in various studies. The objective of this study was to explore the association between MSD and the quality of life and mental health of patients and to discuss issues concerning care seeking patterns in rural Greece.

**Methods:**

Patients registered at one rural Primary Care Centre (PCC) in Crete were invited to complete the Nordic Musculoskeletal Questionnaire (NMQ) for the analysis of musculoskeletal symptoms, together with validated instruments for measuring health related quality of life (SF-36) and mental distress (GHQ-28).

**Results:**

The prevalence rate of MSD was found to be 71.2%, with low back and knee pain being the most common symptoms. Most conditions significantly impaired the quality of life, especially the physical dimensions of SF-36. Depression was strongly correlated to most MSD (*p *< 0.001). Multiple logistic analyses revealed that patients who consulted the PCC due to MSD were likely to have more mental distress or impaired physical functioning compared to those who did not.

**Conclusion:**

Musculoskeletal disorders were common in patients attending the rural PCC of this study and were associated with a poor quality of life and mental distress that affected their consultation behaviour.

## Background

The impact of musculoskeletal disorders (MSD) in the general population has been associated with disability and assessed by measures of health related quality of life (HRQL) [1,2]. HRQL has become an important measure when studying health status and health outcome [3]. Surveys from the industrialized world revealed a high prevalence of MSD and its negative effect on the perceived HRQL, as compared with other common chronic conditions [4]. Musculoskeletal impairments rank number one in chronic impairments in the United States and 1 out of every 4 people in developed and less developed countries reports chronic musculoskeletal pain [5]. As such, the United Nations and WHO declared the decade 2000-2010 as the Bone and Joint Decade with the aim of increasing the understanding of the burden posed by MSD and improving the HRQL of people suffering from them [5].

Several studies within primary care suggest that MSD are a frequent reason for seeking care in primary care. In most European healthcare systems, patients with MSD initially consult a primary care physician, usually a general practitioner (GP) [6]. Care-seeking behaviour due to MSD seems to depend not only on factors associated with the symptoms severity or persistence, but may also be explained by levels of mental distress and depression which have been associated with musculoskeletal pain in various studies [7,8].

However, in Greece, issues regarding the impact of MSD on HRQL remain relatively unexplored. According to a recent study, implemented in rural Crete, the prevalence of any MSD during a 12-month period in patients attending primary care services was reported to be as high as 82.6%, with low back pain being the main complaint. This implied a strong burden for the primary care services [9]. Thus, the current study seeks to examine the impact of musculoskeletal conditions on HRQL and mental health in patients attending a rural primary care setting in Greece. The main objectives of the study are to identify a potential association between MSD, HRQL and mental disorders in a specific primary care population and to investigate the extent to which impaired HRQL and mental disorders affect the consultation rates for MSD patients in primary care.

## Methods

### Setting and sample

Data was collected from one rural Primary Care Centre (PCC) on the island of Crete. This PCC covers a population of approximately 10,000 inhabitants, from a geographically defined area and is staffed by GPs, nurses, midwives, physiotherapists and laboratory technicians who provide primary and emergency care services around the clock. Over a period of 10 working days, all consecutive patients in the waiting room, aged 20-75 years, were eligible to participate in this study. This sample size was sufficient to show statistical significance at 80% for subjects with MSD (a quality of life measure was associated with the presence of MSD if responses to this measure were 50% worse than for subjects without the presence of MSD). The Greek version of the standardised Nordic questionnaire for the analysis of musculoskeletal symptoms (general form) also known as the Nordic Musculoskeletal Questionnaire (NMQ) was used to identify people with musculoskeletal problems [10]. The NMQ is a self-administered questionnaire, designed for the purpose of screening for MSD in epidemiological studies and was translated and validated into the Greek language [11]. The use of NMQ to measure MSD prevalence in a primary care population in Crete has been reported elsewhere [9]. See additional file 1.

### Instruments

Greek versions of the medical outcomes study Short Form 36 measures of health status (SF-36) [12] and the General Health Questionnaire (GHQ-28) [13] were applied to investigate HRQL and mental distress. The SF-36 consists of 8 domains measuring physical functioning, role limitation due to physical health problems, bodily pain, general health, vitality, social functioning, role limitation due to emotional health problems and mental health. Scores are transformed from a range of 0 to 100, with the higher score indicating better HRQL for all domains, except 'bodily pain' where a lower score accounts for less pain and increased quality of life. Physical functioning, role limitation due to physical health problems and bodily pain correlate mainly to physical dimensions, whereas role limitation due to emotional health problems and mental health mostly to mental dimensions of health status. General health, vitality and social functioning correlate to both dimensions. SF-36 has been applied in general population surveys in many countries and has been used for general or specific MSD [14,15]. SF-36 has been validated in the Greek language [16].

The GHQ-28 is a 28-item measure of emotional distress that is divided into four sub-scales: somatic symptoms, anxiety/insomnia, severe depression and social dysfunction. For statistical analysis the binary method of scoring the questionnaire was used. A score of 5 or more in GHQ-28 indicated mental distress. GHQ-28 has also been used to assess psychological distress in surveys of musculoskeletal disorders [17] and has also been validated in the Greek language [18].

Data on height and weight was self-reported via the NMQ and the body mass index (BMI) was calculated. Additional information concerning socio-demographic characteristics (age, gender, cohabitation, education, occupation, clinical co-morbidity and consultations to PCC -to GPs, nurses or physiotherapists) was gathered by reviewing medical records available at the PCC.

### Bioethical committee

At first this study was approved by the Postgraduate Studies Committee of the Medical School of the University of Crete, as a part of a PhD thesis. Then it gained the approval of the Scientific Board of the Regional University Hospital of Heraklion, which serves as a bioethical committee. All the participants were fully informed, through a personal letter of agreement, about the purpose of the study and gave their written consent before the completion of the questionnaires.

### Analysis of data

Cohabitation status was placed into 2 categories (cohabitation/no cohabitation). Educational level and employment status were categorized according to the International Standard Classification [International Standard Classification of Occupations, 88 (ISCO-COM 6) and (International Standard Classification of Education 1997, ISCED 0+1]. Multiple linear regression analyses with backward selection were conducted using physical and mental dimensions of SF-36 as dependent variables and musculoskeletal symptoms, age, gender, education, occupational status and clinical co-morbidity as independent variables. Since both SF-36 and GHQ-28 refer to health indicators during the last few weeks, the estimated 7 days prevalence of musculoskeletal symptoms were used. Regression analysis was performed with GHQ-28 scores as dependent variables to test the possibility of predicting the mental health status from the occurrence of musculoskeletal symptoms. In order to estimate the adjusted odds ratios of the consultations to PCC in relation to scores of SF-36 and GHQ-28 multiple logistic regressions were assessed. Continuous variables are expressed as means (SD). In this analysis the prevalence of pain during the previous 12-months period was used. All analyses were performed using SPSS version 16.0. An a = 0.05 level was set as significant.

## Results

### Participation rate

Participation rate was high (91%), 176 patients (55% females, mean age 54.5 years) agreed to complete the questionnaires. 126 (71.6%) reported at least one musculoskeletal problem during the previous 12-month period (responses to the NMQ questions) with low back pain being the most frequent (n = 76, 43.2%), followed by knee (n = 55, 31.3%), shoulder (n = 53, n = 31.1%) and neck problems (n = 46, 26.1%). Less than half of those who reported MSD (n = 55, 31.3%) attributed to them restrictions in daily activities and 42% reported pain during the previous 7-day period (point prevalence). Women and the elder tended to report more symptoms for every pain site (*p *< 0.05).

### The impact on quality of life

**Figure 1:**
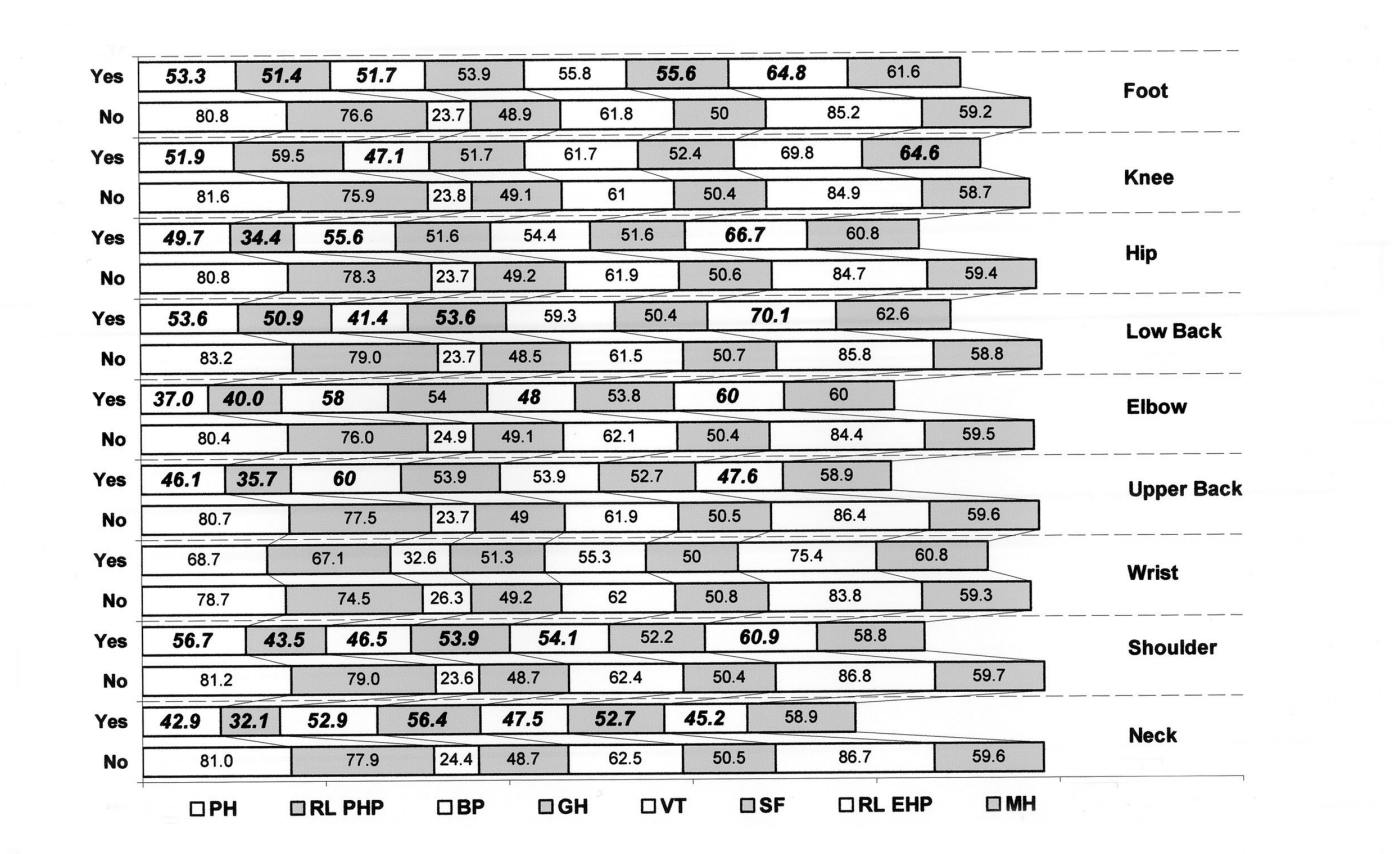
**SF-36 mean scores of patients with and without MSD during the last 7-day period**. Statistical significant values are printed in bold/italics. SD values are presented in the parentheses below. PH: physical functioning (MSD YES: Mean = 30.7, Min = 25.4, Max = 33.6) (MSD NO: Mean = 24.9, Min = 23, Max = 26.3) RL EHP: role limitations due to emotional health problems (MSD YES: Mean = 48.5, Min = 45.7, Max = 51.6) (MSD NO: Mean = 39.3, Min = 37.9, Max = 41.2) BP: bodily pain (MSD YES: Mean = 25.9, Min = 20.4, Max = 32.2) (MSD NO: Mean = 25.3, Min = 24.7, Max = 26.4) GH: general health (MSD YES: Mean = 9.9, Min = 7.8, Max = 13.8) (MSD NO: Mean = 12.1, Min = 11.7, Max = 12.4) VT: vitality (MSD YES: Mean = 19.1, Min = 17.7, Max = 22.5) (MSD NO: Mean = 16.7, Min = 16.4, Max = 17.1) SF: social functioning (MSD YES: Mean = 10.4, Min = 6, Max = 13.1) (MSD NO: Mean = 9.1, Min = 8.8., Max = 9.7) RL PHP: role limitations due to physical health problems (MSD YES: Mean = 46, Min = 38.2, Max = 51.6) (MD NO: Mean = 32.6, Min = 31.1, Max = 34.8) MH: mental health (MSD YES: Mean = 10.4, Min = 9.1, Max = 11.8) (MSD NO: Mean = 10.6, Min = 10, Max = 10.9).

Musculoskeletal symptoms were generally associated with worsened HRQL. Subjects reporting neck pain over the previous 7-day period, had significantly lower scores in SF-36, particularly for physical functioning (SF-36 score, 42.9 versus 81.0, *p *< 0.0001), role limitation due to emotional problems (45.2 v 77.9, *p *< 0.0001), bodily pain (52.9 v 24.4, *p *< 0.001), general health (56.4 v 48.7, *p *= 0.023), vitality (47.5 v 62.5, *p *= 0.002) and role limitation due to physical problems (32.1 v 86.7, *p *< 0.001). Scores relating to other musculoskeletal symptoms are presented in Figure 1 (t-test analysis). Wrist pain did not show a significant effect on any dimension of SF-36. The social functioning, vitality and general health domains demonstrated the least association with MSDs and the mental health domain was affected only by knee pain. Impaired HRQL was particularly evident for the physical functioning, role limitations due to physical health problems and bodily pain domains in patients reporting any musculoskeletal problem comparing with those (n = 50) reporting no musculoskeletal problems at all. In general, HRQL for subjects with coexisting MSD were worse than those with only one disorder. Reporting of more than four musculoskeletal symptoms significantly deteriorated all SF-36 dimensions, except for vitality, social functioning and mental health.

Among subjects reporting musculoskeletal symptoms, age was found to influence physical functioning, role limitations due to physical problems and bodily pain. Obesity and lower education were both related to lower scores in physical functioning. Women reported worst HRQL than men as they scored lower in most SF-36 dimensions, except for vitality, social functioning and mental health. Occupation did not show any significant correlations in the study population. The presence of clinical co-morbidities did not influence the SF-36 scores.

The multiple regression analysis revealed that elderly patients with pain in the hip or the upper back reported more bodily pain. Physical functioning was worse in elderly patients with low back or elbow pain. Role limitations due to physical health problems were worse in overweight patients with hip pain. By summarizing the dimensions of SF-36 into two categories of 'physical dimension' and 'mental dimension', neck pain (Beta = -7.9, 95% C.I. -14.07- -1.93, *p *= 0.01) and upper back pain (Beta = -5.6, 95% C.I. -10.47- -0.68, *p *= 0.02) appeared as the most disabling symptoms, respectively (Table 1).

**Table 1:** Associations between HRQL factors and MSD

Dependent variable	Independent variable	Beta	95% CI	P
**Physical functioning**	Age	-7.49	-11.92-3.06	0.001
	GHQ-28 score	-19.28	-27.21- -11.34	< 0.001
	Elbows pain	-30.53	-47.94- -13.12	0.001
	Low back pain	-19.87	-30.25- -9.49	< 0.001
**Role limitations due to physical health problems**	BMI	-8.21	-16.56-0.14	0.054
	GHQ-28 score	-23.33	-36.89- -9.78	0.001
	Hip pain	-26.70	-48.34- -5.05	0.016
**Bodily pain**	Age	7.21	1.77-12.64	0.010
	GHQ-28 score	8.71	0.51-16.91	0.038
	Hip pain	6.58	5.45-31.51	0.006
	Upper back pain	6.79	11.68-38.55	< 0.001
**General health**	Female Gender	6.38	2.41-10.35	0.002
**Vitality**	GHQ-28 score	-13.07	-19.39- -8.44	< 0.001
	Elbows pain	-12.56	-23.49- -1.62	0.025
**Social functioning**	GHQ-28 score	--3.71	-7.33- - 0.09	0.045
**Role limitations due to emotional health problems**	Female gender	-11.53	-23.04- -0.03	0.049
	GHQ-28 score	-19.97	-32.03 - -7.92	0.001
	Upper back pain	-29.49	-47.30- -11.68	0.001
**Mental health**	GHQ-28 score	-5.06	-8.91 - -1.208	< 0.001
**DIMENSION PHYSICAL**	GHQ-28 score	-9.53	-13.41- -5.66	< 0.001
	Neck pain	-8.00	-14.07- -1.93	0.010
**DIMENSION MENTAL**	GHQ-28 score	-8.50	-11.67- -5.32	< 0.001
	Upper back pain	-5.58	-10.47- -0.68	0.026

Multiple linear regression analysis of SF-36 mean scores and MSD during the previous 7-day period adjusted for age, gender, education level, cohabitation status, GHQ-28 scores and number of co-morbidities. Only the statistically significant correlations are presented.

### The impact on mental health

According to the analysis of GHQ-28 scores, MSD patients were more likely than non-MSD patients to present symptoms of mental distress for every pain reported for the time period of the last week. In multivariate analysis, mental distress, as measured through GHQ-28, added a negative effect in HRQL dimensions of SF-36, except for general health (Table 1). Moreover, patients with mental distress (those who scored positive in GHQ-28) were more likely to be men (Beta = -1.25, 95% C.I. 0.12-0.65, *p *= 0.003) who suffer from neck (Beta = 1.92, 95% C.I. 1.21-38.40, *p *= 0.03) or shoulder pain (Beta = 1.18, 95% C.I. 1.40-7.47, *p *= 0.006), according to the multiple regression analysis.

### The impact on seeking care patterns

Only 32% of those who reported MSD had consulted PCC services during the same period to seek advice on their symptoms. The consultations were referring to GPs, nurses and physiotherapists. Even when participants reported restrictions in their daily activities due to any MSD, they did not consult (crude odds ratio). Mental distress as measured with GHQ-28 (OR = 3.94, 95% C.I. 1.80-8.65, *p *= 0.001), and marginally bodily pain (OR = 1.02, 95% C.I. 1.01 - 1.04, *p *= 0.02) as measured by SF-36, were the main factors affecting a patient with musculoskeletal symptom to consult the PCC (Table 2). Logistic regression analysis revealed significant correlations of the consultations of MSD patients only with physical functioning as measured with SF-36 and depression as measured with GHQ-28.

**Table 2:** Factors affecting the consultations to the PCC

		No consultations		Consultations				
		Number of patients	%	Number of patients	%	*p*	OR	95% C.I.
**GHQ-28**		**<5**	51	77.3	15	22.7		
	**Mental Distress ≥5**	25	46.3	29	53.7	**0.001**	3.94	1.80-8.65
		**Mean scores**	**(SD)**	**Mean scores**	**(SD)**	***p***	**OR**	**95% C.I**
**SF-36**	**Physical Functioning**	79.2	(24.9)	62.4	(31.0)	**0.004**	0.98	0.96-0.99
	**Role Physical**	71.7	(42.5)	61.9	(46.0)	0.261	0.99	0.99-1.00
	**Bodily pain**	27.4	(26,3)	42.7	(23.2)	**0.004**	1.02	1.01-1.04
	**General Health**	50.5	(10.6)	50.9	(14.8)	0.893	1.00	0.97-1.04
	**Vitality**	59.2	(18.2)	60.9	(18.5)	0.639	1.01	0.98-1.03
	**Social Functioning**	50.0	(11.2)	50.9	(9.6)	0.657	1.01	0.97-1.05
	**Role Emotional**	83.8	(34.2)	75.8	(40.6)	0.278	0.99	0.98-1.00
	**Mental Health**	59.2	(11.2)	61.1	(12.7)	0.409	1.01	0.98-1.05
	**DIMENSION Physical**	57.6	(11.2)	55.8	(14.4)	0.452	0.99	0.96-1.02
	**DIMENSION Mental**	60.5	(10.3)	59.9	(11.1)	0.769	0.99	0.96-1.03

Odds Ratio (OR) analysis of scores in GHQ-28 SF-36 associated with consultations to PCC. MSD consultations are referred to the previous 12-months period. Statistical significant correlations are printed in bold.

## Discussion

### Main findings

#### The impact on quality of life

The results of this study demonstrate that people attending primary care services and experiencing MSD have a worse HRQL than those who do not suffer from MSD. The dimensions of physical functioning, role limitations due to physical health problems and bodily pain were the most affected by the presence of MSD, while social functioning, vitality and general health were the least affected. The nature of the musculoskeletal disorders as well as the local social network and cultural traits could explain these findings. Moreover, in agreement with other studies it seems that the physical dimensions were more strongly affected by musculoskeletal symptoms than the psychological dimensions of HRQL [3,15,19]. An interesting finding, according to multiple regression analysis, was that low back pain was not as debilitating as expected, since it was the most common symptom. Possible explanations for this could be that either low back pain is considered a minor symptom, or the study population (rural, self employed as farmers) were able to perform their usual activities, even when experiencing back pain.

Regarding the impact of MSD on HRQL, the literature is inconsistent on the effects of manual/farming activities on the quality of life of the study population, e.g. a study in the UK reports that farmers have greater health needs than non-farmers [20], while another indicates that farmers report less prevalence of psychiatric morbidity [21]. A further study by Saarni et al reports that farmers have poorer working ability and HRQL, but this is not caused by physical health problems [22]. In our study, farming was not significantly related to impaired HRQL in any member of the study population (both MSD and non-MSD patients). Compared with persons without MSD and after controlling for other factors that may interfere with HRQL, only neck and upper back pain were found to have a clearly negative effect on the lives of the affected subjects.

#### The impact on mental health

Our study revealed that people reporting MSD scored higher in GHQ-28 than those without MSD. Depression, anxiety, distress, and related emotions have been related to spinal pain and disability, according to a review of psychological risk factors [23]. Lower education and more mental morbidity were also found to be independently related to sick leave due to neck and low back pain in a rural population [24]. In our study, 42.8% of those who complained of MSD also scored higher in GHQ-28 (≥5), which may constitute a threat to their psychological health. Both of the instruments used, GHQ-28 and SF-36 mental dimensions, seem to be effective in screening for mental disorders in primary care, since their scores were in agreement (r = 0.0381, *p *< 0.001). In addition, 79% of the patients with a known history of depression had scored positive in the GHQ-28.

#### The impact on seeking care patterns

It is apparent from our study that most people with musculoskeletal pain do not seek care from primary care services. In general, factors influencing consultation include patients' demographics, health beliefs and expectations, social status, accessibility to health care, functional status and co-morbidity [25]. According to our findings, patients consulting the PCC due to MSD were more likely to be experiencing mental distress, bad physical functioning and bodily pain. This finding is in agreement with other studies [6]. Pain and lower levels of physical functioning were noticed by primary care consultants for shoulder-neck pain in the UK [26]. The chronicity of pain seems to determine the health care utilization pattern in patients with arthritic pain in the hip or the knee [27]. This finding should be also discussed with caution since other enabling factors, including access to health care services, may interfere. A UK study by Farmer et al states that in general, patients in urban areas consult primary care services more than those in rural areas [28]. Furthermore, a recent study in Greece by Mariolis et al suggests that Greek urban citizens have different health needs, but that back symptoms and muscle pain were the most frequent reasons for patients (aged15-64 years) seeking primary care in the particular rural area studied [29]. Although, MSD patients were more likely to consult the PCC, if they experienced mental distress, it is uncertain whether this could be a characteristic of MSD patients based on the results of this study. This requires further studies and experimental methodology to be clarified.

### Implications of the study

The present findings may have implications both for public health planning and for primary care settings. Physical disability and depression are both predisposing factors for primary care consultations. General practitioners should consider screening for psychological symptoms in all patients with MSD symptoms. Apart from pain and disability management, patients with MSD also need psychological support. GPs need to be adequately trained to deal in a sensitive manner with these patients and their families.

### Strengths and limitations of the study

The small size of the sample raises concerns of type II error and the inclusion of only one PCC raises issues of generalisation of other primary care settings. The fact that 15% of the sample population did not complete the SF-36 may have also introduced a potential bias response. However, non-responders did not significantly differ from responders in terms of age, gender and presence of MSD. Time needed for completion of the questionnaires and place (waiting room in the PCC) was the main issues for non-compliance, according to their reports. Household surveys or pre-arranged telephone interview could overcome this problem.

Although GHQ is not a diagnostic instrument, it can be used in the first stage to identify potential cases of depression, which must in turn be confirmed using clinical assessment [30]. This cross-sectional study reports on the impact of MSD on mental health as measured by a screening scale. Thus, we are unable to comment on whether the identified burden on the affected quality of life reflects the mental health problems experienced or other co-morbidities and conditions which may play a role. Our study cannot explain if mental disorders are a cause or a consequence of MSD factors and further studies are needed.

The fact that the consultation rates were estimated through the medical records may also introduce an information bias, since the credibility of the records was not previously checked. However, the current study contributes to the literature with regard to MSD, mental health and HRQL, by focusing on the relationship between these issues in a primary care setting in a rural location.

## Conclusion

MSD are not only a common cause of physical distress in primary care, but may also co-exist with mental health disorders, worsening further the patients' quality of life. GPs working in primary care settings should be aware of the possible effects of MSD on mental health and quality of life. The current study focused on primary care patients within a rural setting on the Greek island of Crete and research into MSD and quality of life issues are limited within such regions. Thus, local traditions and health beliefs which might affect care seeking behaviour in MSD patients residing in rural areas should also be considered.

## Competing interests

The authors declare that they have no competing interests.

## Authors' contributions

All authors have read and approved the final version of the manuscript. Both CDL and MDA conceived the research idea and prepared the initial manuscript. MDA collected the data of the study. AGH contributed in the manuscript preparation. AKA was in charge for the analysis of the data and participated in the writing of the manuscript.

## Pre-publication history

The pre-publication history for this paper can be accessed here:

http://www.biomedcentral.com/1471-2474/10/143/prepub

## Supplementary Material

Additional file 1 **Greek version of NMQ**. The Greek version of the standardised Nordic questionnaire for the analysis of musculoskeletal symptoms (general form) also known as the Nordic Musculoskeletal Questionnaire (NMQ) is presented. Click here for file
