# Supplementary material for: Studying the association between musculoskeletal disorders, quality of life and mental health. A primary care pilot study in rural Crete, Greece
Source: BMC Musculoskelet Disord. 2009 Nov 20;10:143. doi: 10.1186/1471-2474-10-143 (PMC2785760; doi:10.1186/1471-2474-10-143)
Supplement: Additional file 1 — Greek version of NMQ. The Greek version of the standardised Nordic questionnaire for the analysis of musculoskeletal symptoms (general form) also known as the Nordic Musculoskeletal Questionnaire (NMQ) is presented. [file 1471-2474-10-143-S1.DOC]

| **ΕΝΟΧΛΗΜΑΤΑ ΑΠΟ ΤΟ**  ΕΤΟΣ ΓΕΝΝΗΣΗΣ 1 ΑΝΔΡΑΣ ΑΥΞ. ΑΡΙΘΜΟΣ  **ΜΥΟΣΚΕΛΕΤΙΚΟ ΣΥΣΤΗΜΑ** 19 _ _ 2 ΓΥΝΑΙΚΑ |
| --- |


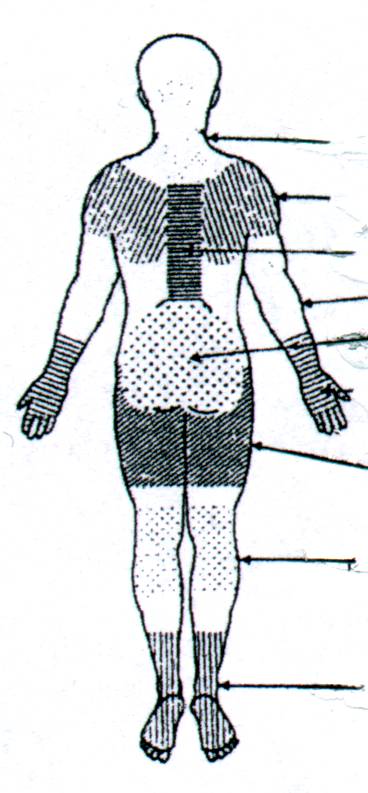


Αυτή η εικόνα δείχνει περίπου, τη θέση εκείνη των

ΑΥΧΕΝΑΣ περιοχών του σώματος που αναφέρονται στο ερωτηματολόγιο.

ΩΜΟΠΛΑΤΙΑΙΕΣ ΠΕΡΙΟΧΕΣ /ΩΜΟΙ Θα πρέπει μόνος σας να αναφέρετε σε ποια περιοχή του

ΑΝΩ ΜΕΡΟΣ ΡΑΧΗΣ σώματός σας εντοπίζονται τα πιθανά ενοχλήματά σας.

ΑΓΚΩΝΕΣ

ΚΑΤΩ ΜΕΡΟΣ ΡΑΧΗΣ Επί πόσα χρόνια και μήνες έχετε τις **^1^**____**^2^**   **^3^** ____**^4^**

ΚΑΡΠΟΙ/ΧΕΡΙΑ τωρινές εργασιακές σας δραστηριότητες ; Χρόνια + μήνες

ΑΚΡΕΣ

ΓΟΦΟΙ Πόσο είναι το εβδομαδιαίο ωράριο σας κατά μέσο όρο ; **^5^**_____**^6^**

ώρες

ΓΟΝΑΤΑ Πόσο ζυγίζετε ; **^7^**_______**^9^**

kg

ΑΣΤΡΑΓΑΛΟΙ/ΠΟΔΙΑ Τι ύψος έχετε; **^10^** _______**^12^** ΑΡΘΡΩΣΕΙΣ cm

**^13^** **1** Δεξιόχειρας **2** Αριστερόχειρας

| **Απαντούνται από όλους** | **Απαντούνται μόνο από τους έχοντες ενοχλήματα** | | |
| --- | --- | --- | --- |
| Είχατε ποτέ ενοχλήματα (πόνος τοπικός ή διάχυτος, δυσφορία) τους τελευταίους 12 μήνες στο/στα : | | Είχατε κάποια φορά κατά τους τελευταίους 12 μήνες πρόβλημα να εκτελέσετε την καθημερινή εργασία σας (εντός ή εκτός σπιτιού) λόγω των ενοχλημάτων ; | Είχατε καθόλου ενοχλήματα τα τελευταία 7 εικοσιτετράωρα ; |
| **^14^ΑΥΧΕΝΑ**  1 Οχι 2 Ναι | | **^15^**  1 Οχι 2 Ναι | **^16^**  1 Οχι 2 Ναι |
| **^17^** **ΩΜΟΠΛΑΤΙΑΙΕΣ ΠΕΡΙΟΧΕΣ/ΩΜΟΥΣ**   1. Οχι 2 Ναι, στη δεξιά ωμοπλατιαία περιοχή/ώμο   3 Ναι, στην αριστερή ωμοπλατιαία περιοχή  /ώμο  4 Ναι, και στις δύο ωμοπλατιαίες  περιοχές/ώμους | | **^18^**  1 Οχι 2 Ναι | **^19^**  1 Οχι 2 Ναι |
| **^20^** **ΑΓΚΩΝΕΣ**  1 Οχι 2 Ναι, στο δεξιό αγκώνα  3 Ναι, στον αριστερό αγκώνα  4 Ναι, και στους δύο αγκώνες | | **^21^**  1 Οχι 2 Ναι | **^22^**  1 Οχι 2 Ναι |
| **^23^** **ΚΑΡΠΟΙ/ΧΕΡΙΑ**  1 Οχι 2 Ναι, στο δεξιό καρπό/χέρι  3 Ναι,στο αριστερό καρπό/χέρι  4 Ναι, και στους δύο καρπούς/χέρια | | **^24^**  1 Οχι 2 Ναι | **^25^**  1 Οχι 2 Ναι |
| **^26^** **ΑΝΩ ΜΕΡΟΣ ΡΑΧΗΣ (θωρακική περιοχή)**  1 Οχι 2 Ναι | | **^27^**  1 Οχι 2 Ναι | **^28^**  1 Οχι 2 Ναι |
| **^29^ ΚΑΤΩ ΜΕΡΟΣ ΡΑΧΗΣ (οσφυική/ιερή περιοχή )**  1 Οχι 2 Ναι | | **^30^**  1 Οχι 2 Ναι | **^31^**  1 Οχι 2 Ναι |
| **^32^** **ΕΝΑ ΓΟΦΟ ή ΚΑΙ ΣΤΟΥΣ ΔΥΟ ΓΟΦΟΥΣ**  1 Οχι 2 Ναι | | **^33^**  1 Οχι 2 Ναι | **^34^**  1 Οχι 2 Ναι |
| **^35^** **ΕΝΑ ΓΟΝΑΤΟ ή ΚΑΙ ΣΤΑ ΔΥΟ ΓΟΝΑΤΑ**  1 Οχι 2 Ναι | | **^36^**  1 Οχι 2 Ναι | ^37^  1 Οχι 2 ΝΑΙ |
| **^38^ ΕΝΑ ΑΣΤΡΑΓΑΛΟ/ΠΟΔΙ ή ΚΑΙ ΣΤΟΥΣ ΔΥΟ**  **ΑΣΤΡΑΓΑΛΟΥΣ/ΠΟΔΙΑ**  1 Οχι 2 Ναι | | **^39^**  1 Οχι 2 Ναι | **^40^**  1 Οχι 2 Ναι |
